# MiST 3.0: an updated microbial signal transduction database with an emphasis on chemosensory systems

**DOI:** 10.1093/nar/gkz988

**Published:** 2019-11-22

**Authors:** Vadim M Gumerov, Davi R Ortega, Ogun Adebali, Luke E Ulrich, Igor B Zhulin

**Affiliations:** 1 Department of Microbiology, The Ohio State University, Columbus, OH 43210, USA; 2 Division of Biology and Biological Engineering, California Institute of Technology, Pasadena, CA 91125, USA; 3 Molecular Biology, Genetics and Bioengineering Program, Faculty of Engineering and Natural Sciences, Sabanci University, Istanbul 34956, Turkey; 4 Ulritech, LLC, Mount Pleasant, SC 29466, USA

## Abstract

Bacteria and archaea employ dedicated signal transduction systems that modulate gene expression, second-messenger turnover, quorum sensing, biofilm formation, motility, host-pathogen and beneficial interactions. The updated MiST database provides a comprehensive classification of microbial signal transduction systems. This update is a result of a substantial scaling to accommodate constantly growing microbial genomic data. More than 125 000 genomes, 516 million genes and almost 100 million unique protein sequences are currently stored in the database. For each bacterial and archaeal genome, MiST 3.0 provides a complete signal transduction profile, thus facilitating theoretical and experimental studies on signal transduction and gene regulation. New software infrastructure and distributed pipeline implemented in MiST 3.0 enable regular genome updates based on the NCBI RefSeq database. A novel MiST feature is the integration of unique profile HMMs to link complex chemosensory systems with corresponding chemoreceptors in bacterial and archaeal genomes. The data can be explored online or via RESTful API (freely available at https://mistdb.com).

## INTRODUCTION

All living organisms need to constantly adapt to changing environmental conditions to ensure survival. The evolutionary success of bacteria and archaea is dependent on the ability of these unicellular organisms to rapidly sense and respond to changes inside and outside their cell. While eukaryotes employ complex signal transduction cascades, bacteria and archaea utilize simpler signal transduction systems. The best studied mode of signal transduction in prokaryotes is two-component signaling (TCS) (1). TCS systems are comprised of two dedicated proteins, a sensor histidine kinase and a dedicated response regulator; some systems contain additional auxiliary components. The most abundant signal transduction systems in prokaryotes, so-called one-component systems (OCS), combine sensory and regulatory functions in a single protein (2). Usually, these functions reside in two distinct domains, sensory and regulatory, although they also can be present in a single domain or distributed between multiple domains within a single protein. Chemosensory systems that involve several dedicated proteins and a specialized version of a histidine kinase comprise the most complex mode of signal transduction in bacteria and archaea (3,4). Functional diversity of signal transduction (linking numerous signals to various types of responses via different modes) is reflected in extreme sequence variation of input and output domains, domain shuffling, and variability of interacting modules, thus presenting a major challenge for genomic identification and logical classification of signal transduction components. The **mi**crobial **s**ignal **t**ransduction (MiST) database was established as a comprehensive signal transduction classification system and has served as a useful community resource since 2007 (5,6).

Exponential growth of DNA sequencing data presents substantial challenges to processing, storing, and retrieving genomic information (7). The MiST 2.0 database (6) was designed to work with the genomic data of a moderate size. It contained 966 complete and 157 draft bacterial and archaeal genomes, which collectively contain >245 000 signal transduction proteins. Currently, the NCBI RefSeq database (Release 95) contains genomes of 58 611 bacterial and archaeal species and hundreds of millions of gene and protein sequences, and these numbers continue to grow. To accommodate a vast amount of ever-growing genomic data, the MiST database has been completely redesigned and rewritten from the ground up. The new software architecture and robust computational pipeline of MiST 3.0 enable efficient processing and storage of constantly growing data. Currently, MiST3 contains >125 000 genomes, 516 million genes, and almost 100 million unique protein sequences. It is the only resource that provides a comprehensive classification of signal transduction in bacterial and archaeal genomes. Microbial two-component signal transduction systems are also classified in the P2CS database (8).

## SIGNAL TRANSDUCTION CLASSIFICATION SCHEMES

Signal transduction proteins and pathways are complex and there is no single, ‘gold standard’ way of classifying them. The current version of MiST utilizes two classification schemes: to categorize signal transduction proteins (provided as ‘Genomic distribution of signal transduction proteins’ tables) and to catalog signal transduction domains (provided as a ‘Signal transduction profile’ graph).

### Classification of signal transduction proteins

For signal transduction proteins, we use the ‘complexity’ scheme – one-component, two-component, and chemosensory systems (2). In addition to classifying pathway components, this scheme allows separating intracellular and extracellular signal transduction pathways: >97% of one-component systems are intracellular sensors and the vast majority of two-component systems contain extracellular sensors (2). The disadvantage of this scheme is that it does not provide a clear separation by protein function for some categories and, in contrast, splits some of the functionally related proteins into different categories. For example, the majority of transcription factors and serine/threonine (ser/thr) kinases would be found in the same category of one-component systems; on the other hand, c-di-GMP-cyclases will be split between the three main categories (one-component, two-component or chemosensory) depending on their associated domains and pathways.

An alternative scheme involves classification by protein function, e.g. placing chemoreceptors, histidine kinases, c-di-GMP-cyclases and phosphodiesterases, ser/thr kinases and other key signal transduction proteins in separate categories (9). While this scheme emphasizes the functional role of a protein, it has its own shortcomings. For example, the same category of response regulators contains such functionally unrelated proteins as transcription factors and c-di-GMP-cyclases, as long as they are associated with the receiver domain. In the case of chemosensory pathways, this scheme splits their components between several categories providing no connection between the elements of the same pathway. We would like to emphasize that our current protein classification scheme does provide functional categorization for several major signal transduction families: chemoreceptors, histidine kinases, response regulators, and extracytoplasmic sigma factors. In the future, we plan to implement an additional classification scheme based exclusively on the protein function, so the users can chose which option to use based on the nature of their inquiry.

### Classification of signal transduction domains

Signal transduction pathways contain various protein domains: many of them are unique to signal transduction, whereas others can play roles in other processes. Here again, there is no simple and unambiguous way for their classification. We present a summary of signal transduction domains for each genome as a graph titled ‘Signal transduction profile’. We classified these domains in seven major categories: (i) input (sensory), (ii) output (regulatory), (iii) chemotaxis (domains specific to chemosensory pathways), (iv) transmitter (transmit information from input), (v) receiver (receive information from transmitter), (vi) ECF and (vii) unknown (any domain, whose role in signal transduction is not understood, but it is found in association with a known signal transduction domain). Input domains are further characterized as (a) cofactor-binding, (b) enzymatic (enzyme-like ligand-binding domains), (c) protein–protein interactions (e.g. domain known to promote protein-protein interactions), (d) signaling (domains associated with signal transduction, but not fully understood), (e) small-ligand binding, and Unknown (the same definition as above). Output domains include the following subcategories: (a) DNA binding (the majority of transcription factors), (b) RNA binding, (c) enzymatic (EAL, GGDEF, Guanylate_cyc domains), (d) protein–protein interactions. Because this scheme classifies domains, not proteins, the same multi-domain protein would appear in various categories. For example, if a protein has a domain, whose role in signal transduction is unknown, and a well-annotated signal transduction domain (e.g. GGDEF), it will be listed both in the ‘unknown' subcategory of the ‘input' category and in the ‘enzymatic' subcategory of the ‘output' category. Systematic exploring of the ‘unknown' subcategories might lead to the discovery of novel signal transduction domains and understanding the roles of other domains in signal transduction. The list of more than 400 protein domains implicated in bacterial and archaeal signal transduction, their annotations and references is provided as Supplementary Table S1 and it is available on the MiST 3.0 Help page, where each domain is hyperlinked with corresponding entries in the Pfam database.

## NEW FEATURES AND IMPROVEMENTS

### Distributed computational pipeline

A vast number of constantly growing bacterial and archaeal genome sequences requires efficient ways to process and store them and imposes strict demands on the hardware. In order to address these challenges we developed a flexible database structure and a distributed computational pipeline, which can run on virtually unlimited number of nodes with a network bandwidth being the only limitation. The pipeline automatically downloads and processes genomes from the NCBI RefSeq database, predicts protein features, identifies and classifies signal transduction proteins, and saves all this data to the MiST database. The signal transduction proteins are classified based on our hierarchical rule system (6) and specific profile hidden Markov models (HMMs). Low-complexity regions and coiled-coils are identified in addition to protein domains as important features for protein function inference. Separate modules that can run independently of each other perform all steps of the pipeline. After installation of Docker (https://www.docker.com/) images, each step of the pipeline runs as a convenient command-line application with very few, well documented, parameters. The pipeline is designed to easily and regularly upload new genomes as they become available and to identify their signal transduction profiles, thus keeping the MiST database up to date.

Although MiST3 is primarily focused on signal transduction, it stores precomputed features of all proteins encoded in NCBI RefSeq genomes. All NCBI RefSeq genomes and the encoded proteins can be explored using convenient features implemented in MiST without running external software. Moreover, proteins of interest can be analyzed in a batch for a set of genomes using the MiST API, which provides many opportunities for large-scale comparative genomic analyses.

### New elaborate database schema

The genomic data is highly linked. To facilitate storage of and interaction with this data, we designed a database structure, which logically distributes all the genomic data in several mutually related tables reflecting their natural relations. The key tables and their relations are listed in Supplementary Table S2. Such a schema allows efficient interaction with the rich genomic data and provides a way to extend the database structure. Understanding the underlying data structure will help researchers to use MiST API most effectively.

### RESTful API

We developed a RESTful API, which allows (i) programmatic access to all the data using a variety of identifiers and parameters and (ii) performing large-scale analysis of bacterial and archaeal signal transduction systems. The requested data is returned in JSON format. A well-documented description of MiST3 data structure together with the detailed query examples in several popular programming languages is given on the API page, which is accessible from https://mistdb.com.

An enormous amount of genomic information prevents returning all the data for a given resource in a single request. For this purpose, any MiST API endpoint that returns an array of records is limited to 30 records per page by default. Query parameters exemplified on the API web page can be used to navigate to subsequent pages and adjust the number of returned records per page.

### New interface

We built a new intuitive web interface for biomedical scientists to explore and analyze bacterial and archaeal genomes. Our new search system allows searching microbial genomes and genes just by typing their identifier without specifying its type. Genomes can be searched by organism name, any taxonomy level (genus, family, *etc*.), RefSeq accession and version, NCBI taxonomy ID and genome assembly level. Genes and proteins can be searched by gene product name, genome locus tags, RefSeq identifier, or using our unique internal stable identifier, which includes the genome RefSeq ID and gene locus. Another new feature of the interface is the ability to filter genomes by taxonomy and assembly level using either embedded filter or selecting corresponding taxonomic name in the drop-down list on the search results table. The genes/proteins search results page contains protein domain information for each returned protein. Genomes and genes can be added to the cart and analyzed in detail and encoded protein sequences can be downloaded. Genomes and genes added to the cart are marked on the search page to help keep track of the added items.

A genome detail page provides comprehensive information about the selected genome including its Bioproject identifier, submitter, and complete description of the signal transduction systems. The signal transduction profile of any given genome is presented as a graph of functional domains together with their counts and a table showing distribution of signal transduction proteins across OCS, TCS and chemosensory systems. The chemosensory systems table shows all the chemosensory pathways encoded in the given genome. Clicking on the graph bars and on the gene counts in the table leads to the list of corresponding signal transduction proteins. Information about a genome analysis state is also provided. A gene/protein detail page contains information about the selected gene, its encoded product, protein domain architecture including details of predicted protein features, and a gene neighborhood graphical representation.

We also implemented a new convenient Scope search which is designed to search for genes and proteins inside a given genome. When a genome name or an identifier is entered in the field called ‘Scope’ on the gene/protein search page, a list of corresponding organisms appears. Clicking on one of them will set it as a genome to search for specified genes and proteins in. The scope can also be set on a genome detail page.

### Novel pathway-specific profile HMMs for accurate classification of chemosensory systems

The Pfam database has a rich collection of profile HMMs for protein domain identification (10). However, its current HMMs do not distinguish between different classes of chemosensory systems and associated chemoreceptors (also known as **m**ethyl-accepting **c**hemotaxis **p**roteins or MCPs). For example, a single HMM (MCPsignal, PF00015) recognizes all MCP classes and there is no specific HMM to recognize the histidine kinase domain of CheA, the central component of the chemosensory system. MCPs are classified in terms of the number of helical heptads that comprise their conserved signaling domain (11). For example, the *E. coli* MCP Tsr has 36 heptads in its signaling domain and thus belongs to the 36H class. Genomic evidence suggested that MCPs of certain heptad classes interact preferentially with certain chemosensory pathway classes defined based on evolutionary considerations (4). Specific profile HMMs were built for nineteen classes of chemosensory pathways (4) and twelve classes of MCPs (11); however, they are not available in Pfam. Furthermore, a new class of signal transduction proteins called MAC (**m**ethyl-**a**ccepting **c**oiled-coil proteins) was identified (4), for which no profile HMM is available. We integrated profile HMMs for different MCP classes, namely 64H, 58H, 52H, 48H, 44H, 42H, 40H, 38H, 36H, 34H, 28H and 24H, into the MiST3 database. We also integrated HMM profiles for components specific to each chemosensory class (CheA, CheB, CheC, CheD, CheR, CheV and CheZ) (4) and newly built profiles for MAC1 and MAC2 protein families. Thus, for the first time, MiST3 offers a comprehensive set of chemosensory pathway-specific HMMs. Using these new profiles in combination with genome neighborhood analysis a complete chemosensory repertoire of any bacterial and archaeal genome can now be reconstructed.

## CASE STUDY

Many bacterial species have multiple chemosensory systems that control several cellular functions (4,12,13). *Pseudomonas aeruginosa* PAO1 has 4 chemosensory pathways encoded by 5 gene clusters and 26 MCPs (14). By searching the MiST3 database, we identified a bacterial genome that encodes the largest number of proteins comprising chemosensory pathways: *Azospirillum* sp. B510 genome (GCF_000010725.1) encodes 126 such proteins, including 88 MCPs. The key question not only in this particular case but in cell biology in general is which receptor feeds into which pathway. Here we show how MiST3 can be used to assign MCPs to chemosensory pathways in a given bacterial genome.

**Figure 1.**
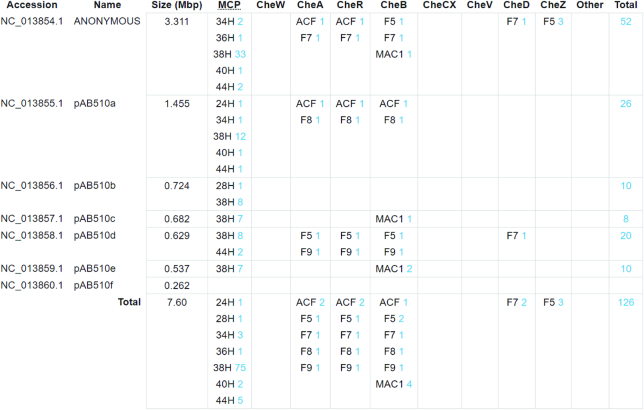
Chemosensory systems table automatically generated by MiST 3.0 for the *Azospirillum* sp. B510 genome.

**Figure 2.**
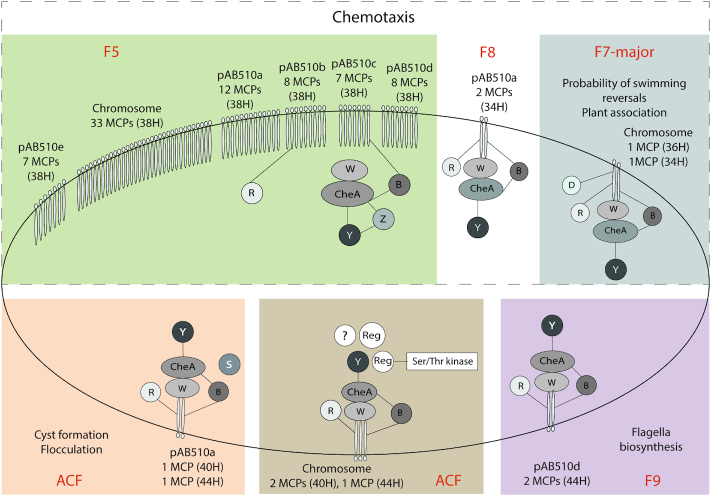
Reconstruction of chemosensory pathways in *Azospirillum* sp. B510 using the MiST 3.0 database.

The MiST3 database automatically identified all components of chemosensory pathways encoded in the *Azospirillum* sp. B510 chromosome and plasmids and assigned them to corresponding classes (Figure 1). In total, six chemosensory pathways were detected: ACF – 2, F7-major – 1, F5 – 1, F8 – 1, and F9 – 1. ACF stands for ‘alternative cellular functions’ and F followed by a number identifies pathways controlling flagella (4). Similarly, all 88 MCPs were assigned to seven heptad classes (24H – 1, 28H – 1, 34H – 3, 36H – 1, 38H – 75, 40H – 2 and 44H – 5). Specific relationships between MCP and chemosensory pathway classes were previously established using large-scale genomic comparisons (4,14). For example, the F7 pathway preferentially utilizes 36H MCPs, whereas the F1 pathway usually contains 44H class MCPs. Using these relationships and gene neighborhood information available in MiST3 we connected all MCPs to corresponding pathways (Figure 2 and Supplementary Table S3). Specific cellular functions can be assigned to four of the six chemosensory pathways (F7-major, F5, F9 and one of the ACF) based on homologous relationships with experimentally studied systems. The F7-major pathway controls chemotaxis in many bacteria, including the model organism *Escherichia coli* (4). The F7-major orthologous cluster in *Azospirillum brasilense* (termed the Che4 cluster) contributes to chemotaxis (15), but this function is also modulated by another chemosensory pathway (termed the Che1 cluster (16)), which is orthologous to the F5 pathway in *Azospirillum* sp. B510. Orthologs of the ACF pathway encoded on pAB510a plasmid control cyst formation in a closely related bacterium *Rhodospirillum centenum* (17) and flocculation in *A. brasilense* (the Che3 cluster) (18). Finally, the F9 ortholog (the Che2 cluster in *A. brasilense*) is involved in controlling flagella biosynthesis in *R. centenum* (19). Taken together, our assignments suggest that the vast majority of MCPs feed into the pathway controlling chemotaxis. Satisfactorily, the same trend was observed in another bacterium with multiple chemosensory systems, *P. aeruginosa* PAO1 (14). While not every MCP was assigned to a specific pathway and some exceptions from the assigned rules are inevitable, this case study demonstrates that MiST3 provides an excellent framework to produce testable hypotheses on bacterial and archaeal signal transduction, and drives future experimental studies.

## AVAILABILITY

The MiST 3.0 database is freely accessible for non-commercial use at https://mistdb.com. Users are not required to register or login to access any feature available in the database. Docker images are available at https://hub.docker.com/u/biowonks/. GitHub repositories locations – https://github.com/biowonks/projects, https://github.com/ToshkaDev/mist-web-v.

## IMPLEMENTATION

The efficiency of the MiST3 database is achieved by using modern technologies on both backend and frontend. The backend and API are implemented using Node.js (https://nodejs.org/en/about/) and Express.js framework (https://expressjs.com/), the frontend was developed using Angular (https://angular.io/). Numerous custom packages were created and used in conjunction with the state-of-the-art vendor packages to interact with the database, process the data, and create images. PostgreSQL is used as a database system. The Docker (https://www.docker.com/) platform was used to provide a reproducible and consistent environment for each component of the application. Protein domains are identified using Pfam (10) profile HMMs running HMMER (20), transmembrane regions – running TM-HMM (21), low-complexity regions – using SEG (22), coiled-coils – using Coils (23), extracytoplasmic function sigma factors – using group-specific profile HMMs and a classification system (24), and signal transduction proteins – using our internal classification procedure based on domain classifications (Supplementary Table S1) and a hierarchical rule system (6).

## CONCLUDING REMARKS

As every resource of this magnitude, MiST remains a work-in-progress platform for exploring signal transduction pathways across >125 000 bacterial and archaeal genomes. Our classification schemes deliver a scalable domain-based categorizing of signal transduction proteins and pathways. The authors would like to state their commitment to maintaining, improving, and updating this database including its classification schemes. MiST3 is open source and we gladly welcome contributions from the community, either as a feature request, bug report or code.

## Supplementary Material

gkz988_Supplemental_Files
