# Supplementary material for: MiST 3.0: an updated microbial signal transduction database with an emphasis on chemosensory systems
Source: Nucleic Acids Res. 2019 Nov 22;48(D1):D459–64. doi: 10.1093/nar/gkz988 (PMC6943060; doi:10.1093/nar/gkz988)
Supplement: gkz988_Supplemental_Files [file gkz988_supplemental_files.zip › Table S2.docx]

| **Table** | **Description** | **References** | **Referenced by** |
| --- | --- | --- | --- |
| Components | Contains the NCBI replicon/contig representation of a given gene or genome including NCBI identifiers. | Genome | Gene,  Signal Genes |
| Genomes | Contains details about genomes including all identifiers, names, source, submitter and metadata. | Components,  has taxonomy id |  |
| Genes | Contains details about genes including all identifiers, product names, location in the genome, corresponding genome identifier and metadata. | Components,  has Aseq id |  |
| Aseqs (1) | Contains predicted protein features and amino acid sequences of all the proteins in the database. |  |  |
| Signal Domains | Contains Pfam superfamilies for signal transduction protein domains. |  | Signal Domains Members |
| Signal Domains Members | Contains representatives of Pfam superfamilies for signal transduction protein domains. | Signal Domains |  |
| Signal Genes | Contains genes from all the genomes in the database involved in signal transduction. | Components,  Genes |  |
| Taxonomy | Contains full taxonomy information for each organism in the database. |  |  |

**Table S2**. Core tables in MiST 3.0 database.

1. Ulrich, L.E. and Zhulin, I.B. (2014) SeqDepot: streamlined database of biological sequences and precomputed features. Bioinformatics, 30, 295-297.
